# Role of steroid minimization in the tacrolimus-based immunosuppressive regimen for liver transplant recipients: a systematic review and meta-analysis of prospective randomized controlled trials

**DOI:** 10.1007/s12072-014-9523-y

**Published:** 2014-03-20

**Authors:** Jinyang Gu, Xingyu Wu, Lei Lu, Shu Zhang, Jianling Bai, Jun Wang, Jun Li, Yitao Ding

**Affiliations:** 1 Department of Hepatobiliary Surgery, Affiliated DrumTower Hospital of Nanjing University Medical School, Nanjing, China; 2 Jiangsu Province’s Key Medical Center for Hepatobiliary Disease, Nanjing, China; 3 Institute of Hepatobiliary Surgery, Nanjing University, Nanjing, China; 4 Department of Surgical Oncology, The 81st Hospital of PLA, Nanjing, China; 5 Department of Gastroenterology, Affiliated DrumTower Hospital of Nanjing University Medical School, Nanjing, China; 6 Department of Epidemiology and Biostatistics, School of Public Health, Nanjing Medical University, Nanjing, China; 7 Department of Hepatobiliary Surgery, DrumTower Clinical Medical College of Nanjing Medical University, Nanjing, China; 8 Department of Ultrasonography, Wuxi Hospital for Infectious Diseases, Wuxi, China

**Keywords:** Liver transplantation, Tacrolimus, Steroid minimization, Randomized controlled trial, Systematic review, Meta-analysis

## Abstract

To evaluate the efficacy and safety of early steroid withdrawal or steroid avoidance in the tacrolimus (Tac)-based immunosuppressive regimen for liver transplant recipients. According to the requirements of the Cochrane systematic review, a thorough literature search was performed in the PubMed/MEDLINE and Cochrane electronic databases between 1995 and 2011 using the key words “liver transplantation,” “Tac,” and “steroid free” or “steroid withdrawal,” restricting articles to the English language. Data were processed for a meta-analysis by Stata 12 software. Altogether 17 prospective randomized controlled trials containing 1,980 transplanted patients were included in this study. The overall pooled RR estimates of 1-, 2-, 3-, and 5-year patient and graft survival rates were 0.985, 0.998, 0.995, and 1.100 (95 % CI 0.925–1.048, 0.934–1.067, 0.894–1.107, and 0.968–1.250, respectively), as well as 0.998, 0.993, 0.945, and 1.053, respectively (95 % CI 0.928–1.072, 0.902–1.092, 0.833–1.072, and 0.849–1.307, respectively). The other pooled RR estimates of acute rejection and chronic rejection rates for all enrolled studies were 1.077 and 0.311 (95 % CI 0.864–1.343 and 0.003–37.207). As for secondary predictors, the pooled RR estimates such as HCV recurrence, HCC recurrence, diabetes, hypertension, kidney dysfunction, bacterial infection, and CMV were 1.101, 1.403, 1.836, 1.607, 0.842, 1.096, and 2.280, respectively (95 % CI 0.964–1.257, 0.422–4.688, 1.294–2.606, 0.926–1.228, 0.693–1.022, 0.783–1.533, and 1.500–3.465, respectively). There were no differences between the steroid group and steroid-free group for all clinical observational indices except for the incidence of diabetes (*p* = 0.001) and CMV infection (*p* < 0.001). In summary, our study indicate that rapid discontinuation of steroid in the Tac-based immunosuppressive regimen may not lead to an increased risk of morbidity and rejection rate.

**Electronic supplementary material:**

The online version of this article (doi:10.1007/s12072-014-9523-y) contains supplementary material, which is available to authorized users.

## Background

Orthotopic liver transplantation (OLT) has been recognized as a well-established therapeutic option for a subset of patients with benign end-stage liver diseases as well as early stage hepatocellular carcinoma (HCC), achieving a favorable long-term survival rate in many liver transplant centers in recent years [1]. It must be admitted that the success of OLT is owed to the pioneers developing the surgical procedures and to the researchers discovering the available medications related to allograft rejection prevention [2]. Although liver allograft is generally considered immunologically privileged, and hyperacute rejection is rarely observed, the substantial short- and long-term morbidity associated with acute and chronic rejection have still set off a wave of investigators seeking a safe and effective immunosuppressive regimen for liver transplant recipients [3, 4].

Steroids have long been recognized as part of the immunosuppressive regimen for induction and maintenance since the advent of clinical OLT [5]. Boluses of high-dose steroids are routinely administered during and after the operation for the control of acute cellular rejection in many liver transplant centers. However, prolonged use of steroids is associated with multiple severe side effects including hypertension, hyperlipidemia, obesity, diabetes mellitus, osteoporosis, infectious complications, and particularly growth retardation in children [6]. In addition, hepatitis C virus (HCV) and tumor recurrence upon OLT should also be taken into consideration when patients are exposed to high-dose long-term steroids [7–10]. In such circumstances, Pirenne et al. reported the long-term (median = 40 months) follow-up data of a prospective study designed to determine whether OLT could be performed with no steroids at all. This prospective single-center pilot study showed that OLT without steroids is feasible and yields no penalty in terms of acute and chronic rejection, immune graft loss, graft function, patient and graft survival [11]. An experience from Germany with about 30 adult liver graft recipients subjected to dual maintenance immunosuppression consisting of tacrolimus (Tac) and mycophenolate mofetil (MMF) without prophylactic steroids revealed that patient and graft survival at 2 years was 86.7 and 83.9 %, respectively [12]. All rejections were completely reversible by temporary addition of steroids. Therefore, the authors speculated that double drug immunosuppression with Tac and MMF is effective and safe in terms of patient and graft survival as well as incidence and severity of rejection [12]. In addition, close drug monitoring is advised after OLT in order to avoid under- or over-immunosuppression, which may be caused by impaired absorption or metabolism [12]. Thus, minimization of steroid usage including steroid-sparing or steroid-free immunosuppressive regimens seems to be the pursued goal for all liver transplant experts to achieve better outcomes [13–16].

However, several pilot studies and a few randomized trials have explored this possibility with mixed results. Reggiani et al. [17] performed a single-center, randomized, 1:1, open-label, controlled study and speculated that a primary immunosuppressive regimen based on Tac and low-dose MMF without steroids is safe but unable to prevent acute rejection at 1 week after transplantation even if early acute rejection does not affect the outcome in terms of morbidity and graft or patient survival. Foroncewicz et al. [18] in Poland conducted a 6-year, single-center, retrospective study including 25 liver transplant recipients. Though results indicated that a steroid-free regimen of Tac is as effective as Tac/steroid in achieving good patient and graft survival, no substantial benefits concerning the safety of Tac therapy were evident during long-term follow-up [18].

More recently, considering the potential detrimental effect on renal functions resulting from the usage of high-dose Tac instead of steroid, some induction agents for specific immunological tolerance including polyclonal rabbit antithymocyte globulin (RATG) and IL-2 receptor monoclonal antibody (basiliximab or daclizumab) have been suggested in triple or quadruple immunosuppressive protocols during OLT, which could minimize the use of Tac and limit renal toxicity [19–21]. To date, there is no consensus about the role of steroid minimization in the Tac-based immunosuppressive regimen for liver transplant recipients. The purpose of our study was to conduct a systematic review and meta-analysis of the published prospective randomized controlled trials since 1995 concerning the efficacy and safety of steroid elimination in a Tac-based immunosuppressive regimen for OLT patients.

## Methods

This is a systematic review including a meta-analysis, which was performed according to the preferred reporting items for the systematic reviews and meta-analyses (PRISMA) statement [22] and the Cochrane Handbook for Systematic Reviews of Interventions [23].

### Search strategy and selection criteria

Two authors (Jinyang Gu and Jun Li) independently searched the databases PubMed/MEDLINE and Cochrane Central Register for all levels of evidence from medical research articles published in print or electronically in English from 1995 to 2011. A global literature search was undertaken by combinations of the following search terms: “liver transplantation,” “Tac,” and “steroid free” or “steroid withdrawal” for the purpose of the role of steroid minimization in the Tac-based immunosuppressive regimen for liver transplant recipients.

The detailed inclusion criteria of trials were as follows: (1) to assure the quality of analysis, only randomized controlled trials were included in the study; (2) comparisons of outcomes were made between a Tac-based immunosuppressive regimen with (lasting time more than 3 months) or without steroid (lasting time within 3 months) for OLT; (3) if multiple publications reported estimates based on the same study population, the largest or most recent sample was used; (4) studies must have reported patient or graft survival rates, acute or chronic rejection prevalence, as well as complication incidence in relation to steroid usage; and (5) our search included only those original articles published in English.

### Data extraction and outcome measures

Two investigators (Jinyang Gu and Jun Li) independently determined the eligibility of each publication for the systematic review and meta-analysis by filling in a Microsoft Excel spreadsheet and evaluating study quality, with disagreements resolved by a third reviewer (Jun Wang). Extracted data included general information (first author, year of publication, study center, and sample size), demographics of participants (gender ratio, mean age, concomitant disease, and MELD score), characteristics of clinical interventions (etiology distribution and immunosuppressive regimen), primary endpoints (survival rates and rejection rates), and secondary endpoints (complication incidences related to steroid usage) from the texts, tables, and graphs of published eligible trial reports. Pooled outcome measures for OLT in a Tac-based immunosuppressive regimen with or without steroid involved patient and graft survival rates at 1, 3, and 5 years, incidence of acute and chronic rejection, incidence of recurrent hepatitis C or HCC, rates of infectious complications, post-OLT metabolic disease occurrence, as well as kidney dysfunction.

### Quality assessment

Two authors (Jinyang Gu and Jun Wang) independently assessed the methodological quality of the included trials using the quality checklist recommended by the Cochrane Handbook [24]. The following domains on the risk of bias were assessed: randomization, patients blinded, concealment of treatment allocation, intention-to-treat analysis, and incomplete outcome. We resolved all disagreements by discussion and referral to a third author (Jun Li) for adjudication.

### Data synthesis and analysis

We processed data in accordance with the Cochrane Handbook for Systematic Reviews of Interventions [23]. Funnel plots and Egger’s tests were created using standard techniques for detecting publication bias. For randomized controlled trials, outcome data were pooled using a random effect model weighted by the inverse variance. The meta-analyses results of continuous variables were expressed as mean differences and as risk ratios (RR) for binary outcomes with 95 % CI. Meta-analyses of the binary variables were conducted on the log-odds ratios to satisfy the assumption of normality of effect sizes. Statistical analyses were performed using STATA 12. Instead, we undertook specific stratified meta-analyses to examine the sensitivity of the findings of the review to key potential causes of heterogeneity.

### Publication bias

We assessed the potential for publication bias through visual inspection of funnel plot asymmetry and evaluated the statistical significance of differences among the including trials with Begg’s test.

## Results

### Literature search results

**Fig. 1:**
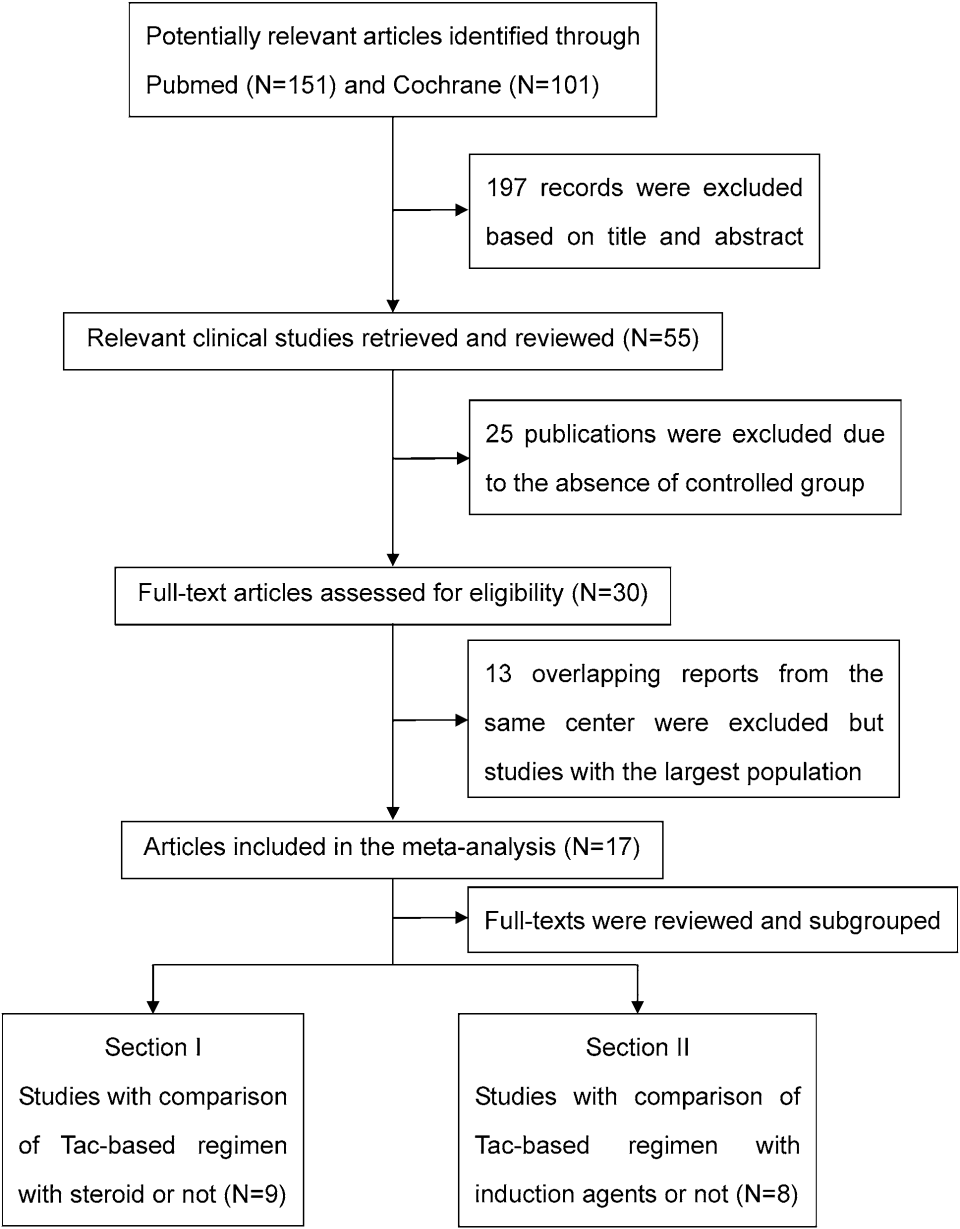
Diagram of the literature search and selection process. A total of 252 citations comprising 151 publications in PubMed and 101 in the Cochrane Central Register were yielded between 1995 and 2011. We identified 55 potentially relevant studies that were retrieved and reviewed by titles and abstracts, 25 of which were further excluded because of the absence of a control group or lack of a detailed outcome index. Of the 30 possible studies meeting our inclusion criteria, 13 duplicate papers deriving from the same clinical centers were excluded, and finally 17 eligible full-text articles were included with the largest population and distinct observational index in this meta-analysis, which were further divided into two sections consisting of studies with comparison of Tac-based immunosuppressive regimens with steroids or not, as well as with induction agents or not

The electronic database searches yielded a total of 252 citations comprising 151 publications in PubMed and 101 in the Cochrane Central Register between 1995 and 2011. We identified 55 potentially relevant studies that were retrieved and reviewed by titles and abstracts, 25 of which were further excluded because of the absence of a control group or lack of a detailed outcome index. Of the 30 possible studies meeting our inclusion criteria, 13 duplicate papers derived from the same clinical centers were excluded from our present study, and we finally included 17 eligible full-text articles with the largest population and distinct observational index in this meta-analysis, which were further divided into two sections consisting of studies with comparison of Tac-based immunosuppressive regimens with steroids or not, as well as with induction agents or not. The flow chart of the search and selection is illustrated in Fig. 1.

### Characteristics of included studies

Study and patient characteristics are summarized in Tables 1 and 2. Overall, the 17 randomized, controlled trials enrolled a total of 1,980 participants with a mean age of 44.4 years, of which approximately 65 % were male. Five studies were based in the USA [26, 32, 35, 36, 38], three in Germany [25, 28, 31], three in Italy [17, 29, 34], and one each in Spain [27], China [9], UK [30], France [33], Belgium [37], and Poland [18]. The 17 prospective randomized controlled studies enrolled patients with distinct primary diseases eligible for OLT such as hepatitis B virus (HBV) [18, 27, 31–33], HCV infection [18, 25–27, 30–33, 35, 36, 38], HCC [9, 17, 26, 27, 30, 31, 33, 37], primary sclerosing cholangitis (PSC) or primary biliary cirrhosis (PBC) [17, 18, 31, 32], alcoholic cirrhosis [17, 18, 26, 27, 31], and autoimmune hepatitis (AIH) [28, 32]. A proportion of selected studies (6/17) described concomitant diseases such as diabetes [27, 29–31, 33], hypertension [27, 31], cytomegalovirus (CMV) infection [33, 34], Epstein-Barr virus (EBV) infection [33, 34], and metabolic disease [31, 33, 34, 37]. Of these 17 studies, all but 2 publications [28, 30] reported intraoperative steroid usage to avoid hyperacute rejection. As far as postoperative steroid duration was concerned, it was totally different among liver transplantation centers ranging from 3 to 72 months for the control group (steroid group) and <3 months for the experimental group (steroid-free group). The remnant observational index including time of Tac and MMF duration, and Tac blood level is displayed in detail in Table 2. The overall 17 prospective randomized controlled trials were then divided into two parts in terms of whether steroid was employed upon OLT or not (Sect. I), as well as whether induction agents were employed during OLT (Sect. II), which was further analyzed for all 17 trials and for each section, respectively. Tables 3 and 4 display a summary of outcomes including survival rates and complication incidence.

**Table 1:** Patient demographics and baseline characteristics of 17 enrolled RCTs in this meta-analysis

First author	Year	Study center	Group	No of patients (*n*)	Gender (M/F)	Mean age (year)	Concomitant disease (*n*)					MELD score
							Diabetes	Hypertension	CMV	EBV	Metabolic disease	
Comparison of Tac-based regimen with steroid or not (Sect. I)												
Langrehr [25]	2002	University of Berlin, Germany	Tac + steroid	15								
			Tac + MMF	15								
Pelletier [26]	2005	University of Michigan, USA	Tac + MMF + steroid	36	28/8	53.0						18.0
			Tac + MMF	36	25/11	55.0						17.0
Margarit [27]	2005	Universidad Autònoma Barcelona, Spain	Tac + steroid	32	25/7	56.0	5	6				
			Tac	28	18/10	57.0	6	3				
Reggiani [17]	2005	Instituto di Ricovero e Cura a Caraterre Scientifico (IRCCS) Policlinico San Matteo, Italy	Tac + MMF + steroid	18	13/5	50.4						
			Tac + MMF	12	8/4	49.7						
Junge [28]	2005	Charité Berlin Campus Virchow Klinikum, Germany	Tac + steroid	14								
			Tac + MMF	16								
Chen [9]	2007	Tongji Medical College, China	Tac + MMF + steroid	26	0/26	47.4						
			Tac + MMF	28	1/27	45.7						
Vivarelli [29]	2007	University of Bologna, Italy	Tac + steroid	16		58.9	5					16.0
			Tac	23		57.2	7					15.0
Manousou [30]	2009	University College London, UK	Azathioprine + steroid + Tac	49		50.0	13					
			Tac	54		48.9	13					
Weiler [31]	2010	Hospital of Johannes Gutenberg University Mainz, Germany	Tac + steroid	54	36/18	53.5	10	10			0	
			Tac	56	38/18	53.6	11	6			2	
Comparison of Tac-based regimen with induction agents or not (Sect. II)												
Eason [32]	2003	Ochsner Clinic Foundation, New Orleans, USA	Tac + MMF + steroid	59								
			RATG + Tac + MMF	60								
Boillot [33]	2005	Hospital Edouard Herriot, France	Tac + steroid	347	238/109	51.0	55		248	237	2	
			Daclizumab + Tac	351	239/112	50.9	57		240	236	7	
Spada [34]	2006	University of Pittsburgh Medical Center, Italy	Tac + steroid	36	15/21	2.8			20	11	3	
			Basiliximab + Tac	36	18/18	2.9			19	12	2	
Humar [35]	2007	University of Minnesota Minneapolis, USA	Tac + MMF + steroid	83		51.8						23.0
			Basiliximab + Tac + MMF	83		52.3						28.0
Kato [36]	2007	University of Miami School of Medicine, USA	Tac/MMF + steroid	39	29/10	50.2						16.5
			Daclizumab + Tac/MMF	31	21/10	52.4						14.6
Gras [37]	2008	Luc University Clinics, Université Catholique de Louvain, Belgium	Tac + steroid	34	16/18	2.0					4	
			Basiliximab + Tac	50	27/23	1.7					4	
Foroncewicz [18]	2009	Medical University of Warsaw, Poland	Tac + steroid	18	10/8	41.8						
			Daclizumab + Tac	7	5/2	43.3						
Klintmalm [38]	2011	Baylor University Medical Center, USA	Tac + MMF + steroid	72	54/18	51.6						
			Daclizumab + Tac + MMF	146	105/41	51.3						

*CMV* cytomegalovirus, *EBV* Epstein-Barr virus, *MELD* model for end-stage liver disease, *MMF* mycophenolate mofetil, *RATG* rabbit antithymocyte globulin, *Tac* tacrolimus

**Table 2:** Main characteristics of clinical interventions in Tac-based immunosuppressive regimens during liver transplantation

First author	Year	Study center	Group	No of patients (*n*)	Etiology distribution (*n*)						Intraoperative steroid usage	Postoperative steroid duration	Tac duration	MMF duration	Tac blood level (ng/mL)		
					HBV	HCV	HCC	PSC/PBC	Alcohol	AIH					7 day	28 day	90 day
Comparison of Tac-based regimen with steroid or not (Sect. I)																	
Langrehr [25]	2002	University of Berlin, Germany	Tac + steroid	15		15					+	14 m					
			Tac + MMF	15		15					+	One bolus					
Pelletier [26]	2005	University of Michigan, Ann Arbor, USA	Tac + MMF + steroid	36		24	8		13		+	6 m	14.0 m	14 m			
			Tac + MMF	36		14	6		17				14.0 m	14 m			
Margarit [27]	2005	Universidad Autònoma Barcelona, Spain	Tac + steroid	32	2	15	11		11		+	3 m	44.0 m		12.0	13.0	10.0
			Tac	28	3	20	13		5		+		44.0 m		12.5	14.0	10.5
Reggiani [17]	2005	Instituto di Ricovero e Cura a Caraterre Scientifico (IRCCS) Policlinico San Matteo, Italy	Tac + MMF + steroid	18			12	1	1		+	3 m	31.0 m	31 m	11.0	12.0	
			Tac + MMF	12			2	0	2				31.0 m	31 m	14.8	11.0	
Junge [28]	2005	Charité Berlin Campus Virchow Klinikum, Germany	Tac + steroid	14						14		3 m	24.0 m	24 m			
			Tac + MMF	16						16			24.0 m	24 m			
Chen [9]	2007	Tongji Medical College, China	Tac + MMF + steroid	26			26				+	12 m	12.0 m	12 m			
			Tac + MMF	28			28				+	3 m	12.0 m	12 m			
Vivarelli [29]	2007	University of Bologna, Italy	Tac + steroid	16							+	24 m	28.0 m		11.2	11.5	8.0
			Tac	23							+	3 m	28.0 m		12.0	11.1	9.1
Manousou [30]	2009	University College London, UK	Azathioprine + steroid + Tac	49		49	13					6 m	53.5 m			8.4	7.0
			Tac	54		54	17						53.5 m			8.0	8.0
Weiler [31]	2010	Hospital of Johannes Gutenberg University Mainz, Germany	Tac + steroid	54	7	16	19	5	21		+	6 m	60.0 m				
			Tac	56	12	14	21	3	16		+	2w	60.0 m				
Comparison of Tac-based regimen with induction agents or not (Sect. II)																	
Eason [32]	2003	Ochsner Clinic Foundation, USA	Tac + MMF + steroid	59	1	34		6		3	+	3 m	18.0 m	3 m			
			RATG + Tac + MMF	60	3	31		3		3			18.0 m	3 m			
Boillot [33]	2005	Hospital Edouard Herriot, France	Tac + steroid	347	55	103	50				+	3 m	3.0 m				10.9
			Daclizumab + Tac	351	63	106	53				+		3.0 m				10.6
Spada [34]	2006	University of Pittsburgh Medical Center, Italy	Tac + steroid	36							+	6 m	24.0 m		7.8		9.3
			Basiliximab + Tac	36							+		24.0 m		9.9		7.5
Humar [35]	2007	University of Minnesota Minneapolis, USA	Tac + MMF + steroid	83		42					+	6 m	32.0 m	3 m			
			Basiliximab + Tac + MMF	83		44					+	6d	16.1 m	3 m			
Kato [36]	2007	University of Miami School of Medicine, USA	Tac/MMF + steroid	39		39					+	3 m	52.0 m	12 m			
			Daclizumab + Tac/MMF	31		31							52.0 m	12 m			
Gras [37]	2008	Luc University Clinics, Université Catholique de Louvain, Belgium	Tac + steroid	34			4				+	60 m	60.0 m		11.8	8.8	7.7
			Basiliximab + Tac	50			8						60.0 m		9.9	9.0	6.9
Foroncewicz [18]	2009	Medical University of Warsaw, Poland	Tac + steroid	18	2	2		5	2		+	72 m	72.0 m				
			Daclizumab + Tac	7	0	3		2	1		+		72.0 m				
Klintmalm [38]	2011	Baylor University Medical Center, USA	Tac + MMF + steroid	72		72					+	20.9 m	20.9 m	20.9 m	11.1	11.1	11.1
			Daclizumab + Tac + MMF	146		146							20.9 m	20.9 m	10.8	11.1	9.6

*AIH* autoimmune hepatitis, *HBV* hepatitis B virus, *HCV* hepatitis C virus, *HCC* hepatocellular carcinoma, *MMF* mycophenolate mofetil, *PBC* primary biliary cirrhosis, *PSC* primary sclerosing cholangitis, *RATG* rabbit antithymocyte globulin, *Tac* tacrolimus

**Table 3:** Summary of primary endpoints including survival rates and rejection rates in this study

First author	Average follow-up period	Follow-up completeness (%)	Group	No. of patients (*n*)	Patient survival (*n*)				Graft survival (*n*)				Acute rejection (*n*)	Chronic rejection (*n*)
					1 year	2 year	3 year	5 year	1 year	2 year	3 year	5 year		
Comparison of Tac-based regimen with steroid or not (Sect. I)														
Langrehr [25]	425 days	100	Tac + steroid	15	14	14							7	
			Tac + MMF	15	14	14							4	
Pelletier [26]	412 days	100	Tac + MMF + steroid	36	32				32				5	
			Tac + MMF	36	30				29				9	
Margarit al. [27]		100	Tac + steroid	32	27		25	23	24		19	19	10	0
			Tac	28	24		23	18	24		23	18	11	0
Reggiani al. [17]	31 months	100	Tac + MMF + steroid	18		18				15			3	
			Tac + MMF	12		11				11			9	
Junge [28]	67 months	100	Tac + steroid	14		13							6	1
			Tac + MMF	16		16							6	0
Chen [9]		100	Tac + MMF + steroid	26	12								3	
			Tac + MMF	28	18								4	
Vivarelli [29]	841 days	100	Tac + steroid	16		12							4	
			Tac	23		18							2	
Manousou [30]	53.5 months	100	Azathioprine + steroid + Tac	49				43					31	
			Tac	54				45					22	
Weiler [31]		100	Tac + steroid	54	48	46	44	43	44	43	42	39	14	0
			Tac	56	48	44	43	39	47	41	40	36	19	16
Comparison of Tac-based regimen with induction agents or not (Sect. II)														
Eason [32]	18.5 months	100	Tac + MMF + steroid	59	50	49			47				18	
			RATG + Tac + MMF	60	51	49			49				15	
Boillot [33]		100	Tac + steroid	347									92	
			Daclizumab + Tac	351									89	
Spada [34]		100	Tac + steroid	36	33	33			31	31			11	
			Basiliximab + Tac	36	32	32			29	29			4	
Humar [35]	24 months	100	Tac + MMF + steroid	83	67				67				10	
			Basiliximab + Tac + MMF	83	73				71				9	
Kato [36]		100	Tac/MMF + steroid	39	31				31				17	
			Daclizumab + Tac/MMF	31	23				21				10	
Gras [37]		100	Tac + steroid	34			31				30			
			Basiliximab + Tac	50			48				47			
Foroncewicz [18]	6 years	100	Tac + steroid	18									4	
			Daclizumab + Tac	7									1	
Klintmalm [38]	20.9 months	100	Tac + MMF + steroid	72		58				57			7	
			Daclizumab + Tac + MMF	146		126				124			17	

*MMF* mycophenolate mofetil, *RATG* rabbit antithymocyte globulin, *Tac* tacrolimus

**Table 4:** Summary of secondary endpoints including complication incidences related to steroid usage in this study

First author	Year	Study center	Group	No. of patients (*n*)	HCV recurrence (*n*)	HCC recurrence (*n*)	Diabetes (*n*)	Hypertension (*n*)	Kidney dysfunction (*n*)	Bacterial infection (*n*)	CMV (*n*)
Comparison of Tac-based regimen with induction agents or not (Sect. I)											
Langrehr [25]	2002	University of Berlin, Germany	Tac + steroid	15	3					2	
			Tac + MMF	15	2					0	
Pelletier [26]	2005	University of Michigan, Ann Arbor, USA	Tac + MMF + steroid	36	17					10	
			Tac + MMF	36	18					19	
Margarit [27]	2005	Universidad Autònoma Barcelona, Spain	Tac + steroid	32	20	0	6	3	17	30	3
			Tac	28	11	1	2	1	20	26	0
Reggiani [17]	2005	Instituto di Ricovero e Cura a Caraterre Scientifico (IRCCS) Policlinico San Matteo, Italy	Tac + MMF + steroid	18			5	5	3	5	
			Tac + MMF	12			2	2	2	6	
Junge [28]	2005	Charité Berlin Campus Virchow Klinikum, Germany	Tac + steroid	14			6				
			Tac + MMF	16			2				
Chen [9]	2007	Tongji Medical College, China	Tac + MMF + steroid	26		18					
			Tac + MMF	28		11					
Vivarelli et al. [29]	2007	University of Bologna, Italy	Tac + steroid	16	15		7				
			Tac	23	22		7				
Manousou [30]	2009	University College London, UK	Azathioprine + steroid + Tac	49	10		14		19		10
			Tac	54	17		15		20		7
Weiler [31]	2010	Hospital of Johannes Gutenberg University Mainz, Germany	Tac + steroid	54	6		12	31			
			Tac	56	10		18	36			
Comparison of Tac-based regimen with induction agents or not (Sect. II)											
Eason [32]	2003	Ochsner Clinic Foundation, USA	Tac + MMF + steroid	59	24		8			13	14
			RATG + Tac + MMF	60	18		1			15	3
Boillot [33]	2005	Hospital Edouard Herriot, France	Tac + steroid	347			53	50	82	39	40
			Daclizumab + Tac	351			20	45	98	39	18
Spada [34]	2006	University of Pittsburgh Medical Center, Italy	Tac + steroid	36	8			8	1	30	
			Basiliximab + Tac	36	7			3	4	14	
Humar [35]	2007	University of Minnesota Minneapolis, USA	Tac + MMF + steroid	83	46		27				1
			Basiliximab + Tac + MMF	83	32		10				1
Kato [36]	2007	University of Miami School of Medicine, USA	Tac/MMF + steroid	39	6		14	17		12	
			Daclizumab + Tac/MMF	31	4		3	10		2	
Gras [37]	2008	Luc University Clinics, Université Catholique de Louvain, Belgium	Tac + steroid	34						34	
			Basiliximab + Tac	50						50	
Foroncewicz [18]	2009	Medical University of Warsaw, Poland	Tac + steroid	18			4			10	
			Daclizumab + Tac	7			1			3	
Klintmalm [38]	2011	Baylor University Medical Center, USA	Tac + MMF + steroid	72	55		24	51			
			Daclizumab + Tac + MMF	146	99		27	97			

*CMV* cytomegalovirus, *HBV* hepatitis B virus, *HCV* hepatitis C virus, *HCC* hepatocellular carcinoma, *MMF* mycophenolate mofetil, *RATG* rabbit antithymocyte globulin, *Tac* tacrolimus

### Quality assessment

We evaluated the quality of each trial according to the Jadad scale. Five domains were assessed: randomization, patient blinding, concealment of treatment allocation, intention-to-treat analysis, and incomplete outcome (Supplementary Table 1). All included articles described their study design as prospective randomized controlled trials. Only 11.8 % (2/17) reported patient blinding and concealed allocation [30, 31], and 23.5 % (4/17) used intention-to-treat analysis [26, 30, 33, 38]. In addition, the overwhelming majority of publications lacked complete outcome data except for two [27, 31].

#### Primary predictors

**Fig. 2:**
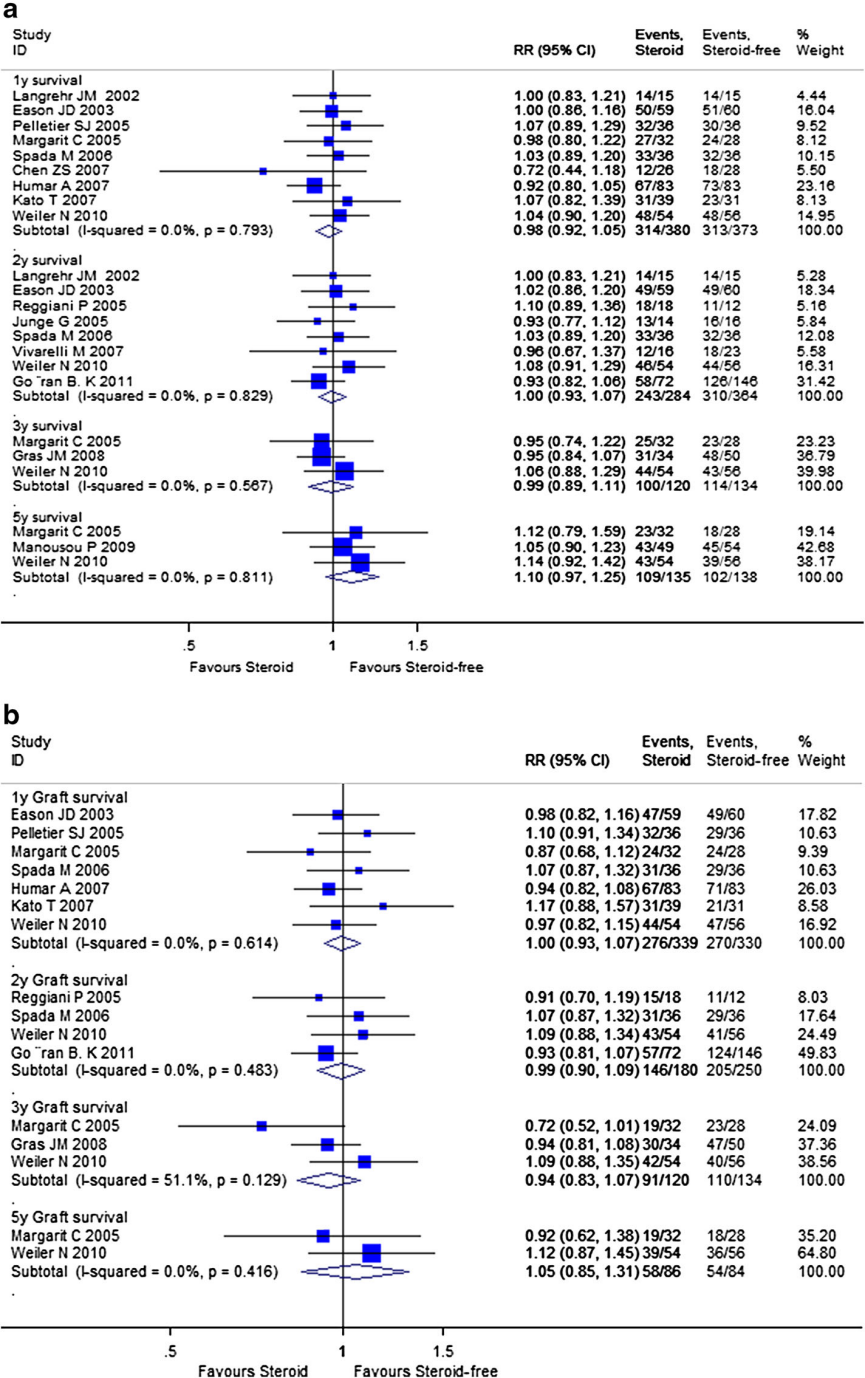

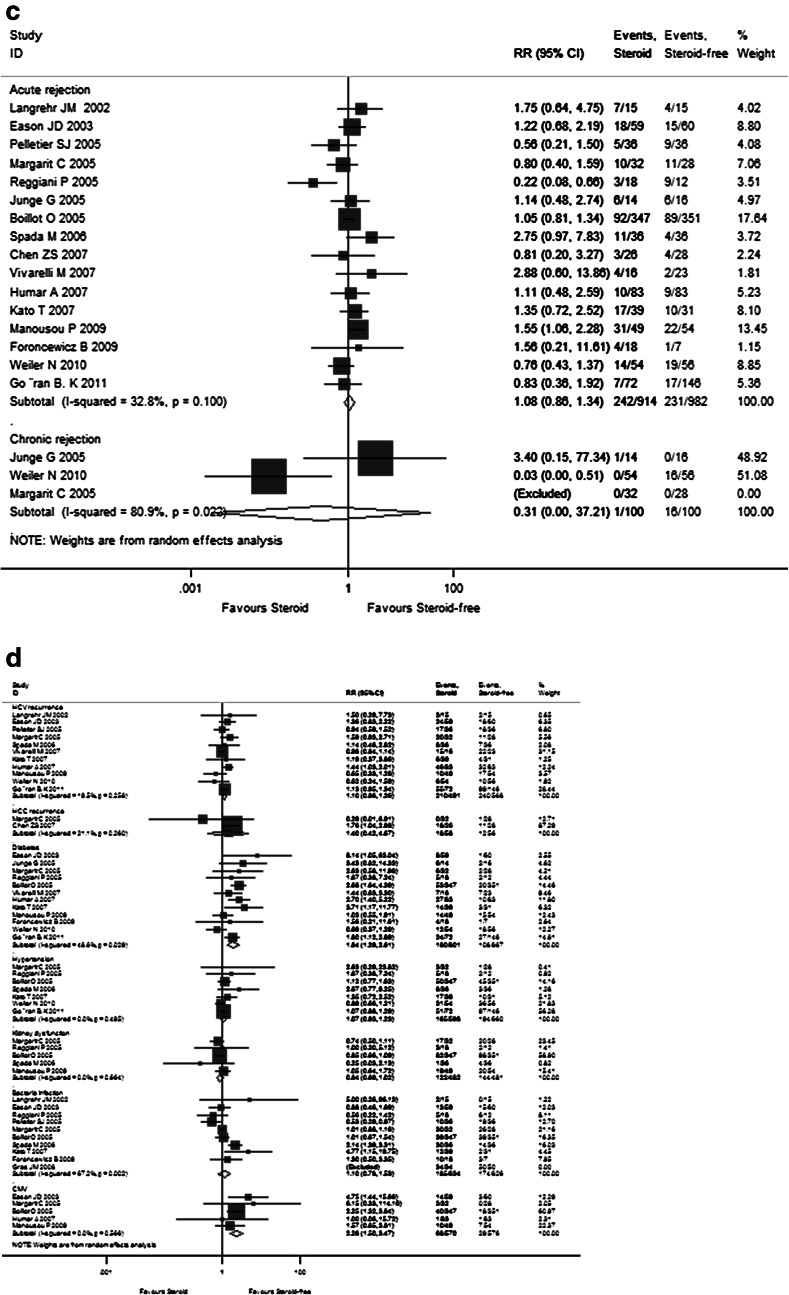
Forest plot of RR and 95 % CI for patient survival rates (**a**), graft survival rates (**b**), and rejection rates (**c**) and incidence of complications (**d**) for all 17 enrolled RCTs in this study. The *horizontal lines* represent the 95 % CI of the RR for the steroid group compared to steroid-free group in each study. The *black box* in the middle of the CI represents the single best estimate of RR in that study. The width of the CI is related to the power of the study and inversely associated with sample size. In addition, the pooled or combined RR results of the meta-analysis are represented by a *diamond*, the width of which is the CI for the pooled data. The *vertical line* is typically displayed to indicate no effect when RR = 1. When the CI crosses the *vertical line* of no effect, we must accept the null hypothesis of no difference between two groups. Only if the CI remains clear of the *vertical line* of no effect can we reject the null hypothesis and conclude that steroid minimization likely caused the outcome. We used a fixed effect model for meta-analysis, except that heterogeneity between studies was considered present if the *p* value was <0.1 or *I* ^*2*^ was more than 50 %, where we used a random effect model instead

Supplementary Table 2 summarizes the meta-analysis results of pooled primary outcomes for all 17 enrolled RCTs in this study. The overall 1-, 2-, 3-, and 5-year pooled RR estimates of patient survival rates and graft survival rates were 0.985 (95 % CI 0.925–1.048), 0.998 (95 % CI 0.934–1.067), 0.995 (95 % CI 0.894–1.107), 1.100 (95 % CI 0.968–1.250), as well as 0.998 (95 % CI 0.928–1.072), 0.993 (95 % CI 0.902–1.092), 0.945 (95 % CI 0.833–1.072), and 1.053 (95 % CI 0.849–1.307), respectively (Fig. 2a, b). The other pooled RR estimates of acute rejection and chronic rejection rates for all enrolled studies were 1.077 (95 % CI 0.864–1.343) and 0.311 (95 % CI 0.003–37.207) (Fig. 2c). There were no differences between the steroid group and steroid-free group for the primary endpoints.

**Fig. 3:**
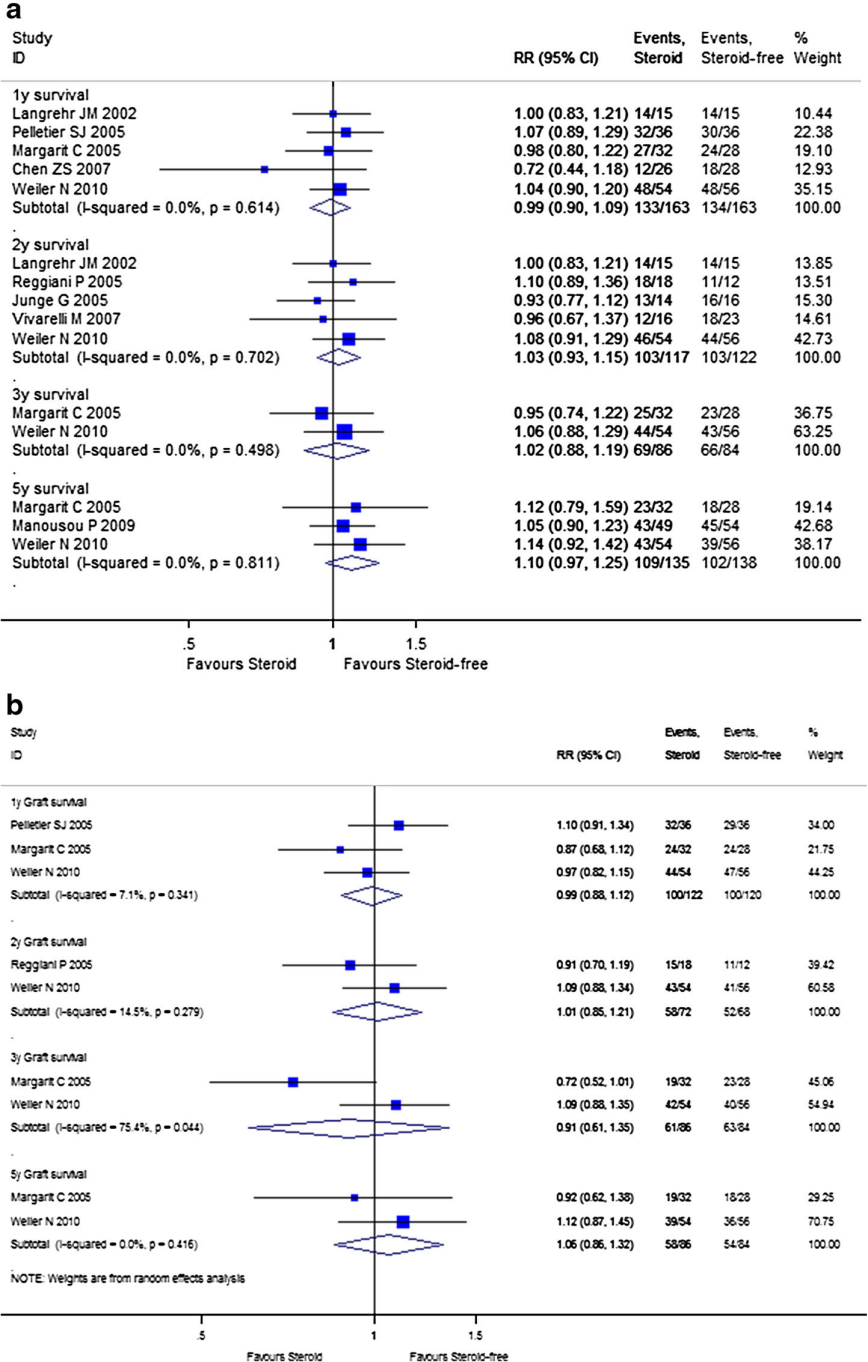

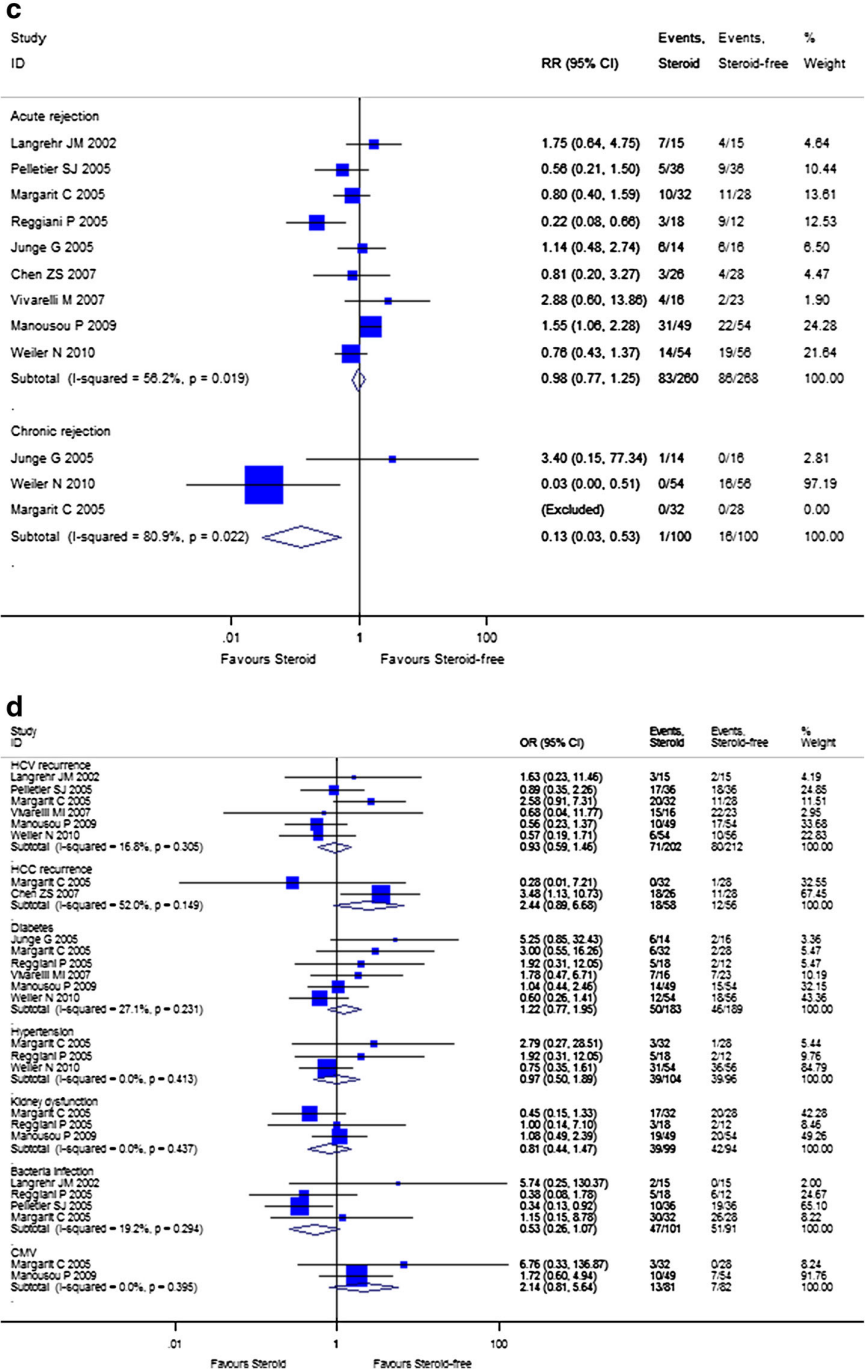
Forest plot of RR and 95 % CI for patient survival rates (**a**), graft survival rates (**b**), and rejection rates (**c**) and incidence of complications (**d**) for Sect. I in this study. The *horizontal lines* represent the 95 % CI of the RR for the steroid group compared to the steroid-free group in each study. The *black box* in the middle of the CI represents the single best estimate of RR in that study. The width of the CI is related to the power of the study and inversely associated with sample size. In addition, the pooled or combined RR results of the meta-analysis are represented by a *diamond*, the width of which is the CI for the pooled data. The *vertical line* is typically displayed to indicate no effect when RR = 1. When the CI crosses the *vertical line* of no effect, we must accept the null hypothesis of no difference between two groups. Only if the CI remains clear of the *vertical line* of no effect can we reject the null hypothesis and conclude that steroid minimization likely caused the outcome. We used a fixed effect model for meta-analysis, except that heterogeneity between studies was considered present if the *p* value was <0.1 or *I* ^*2*^ was more than 50 %, where we used a random effect model instead

**Fig. 4:**
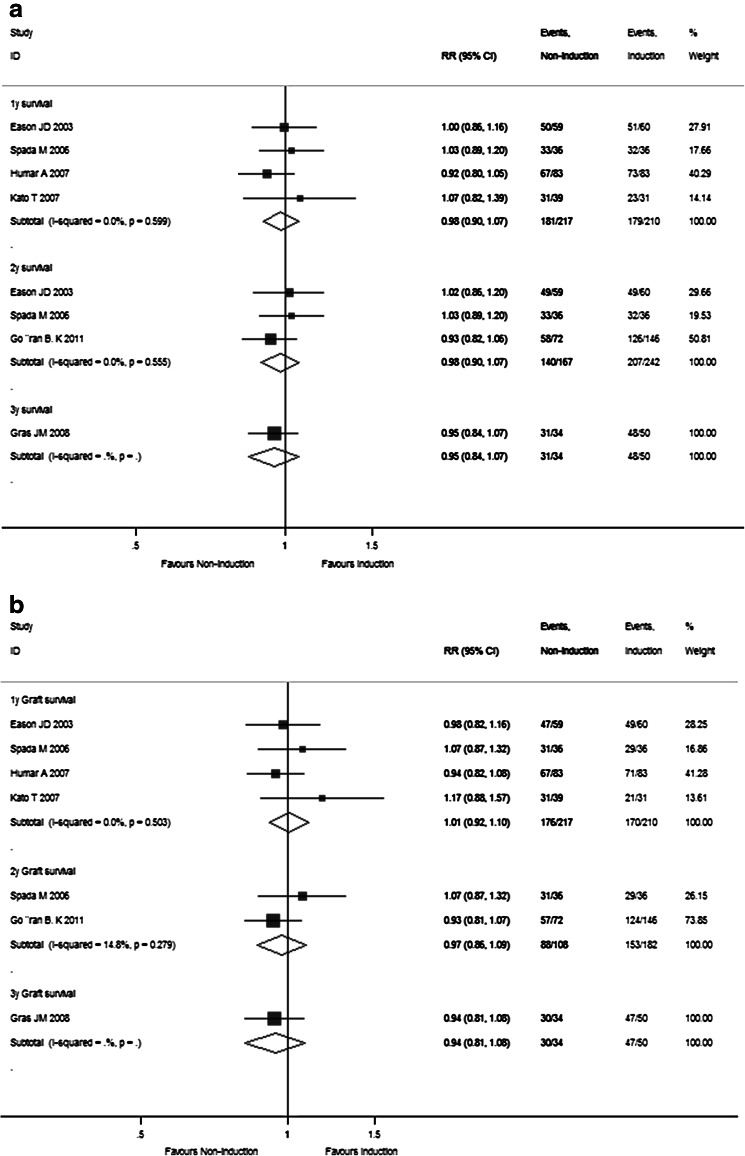

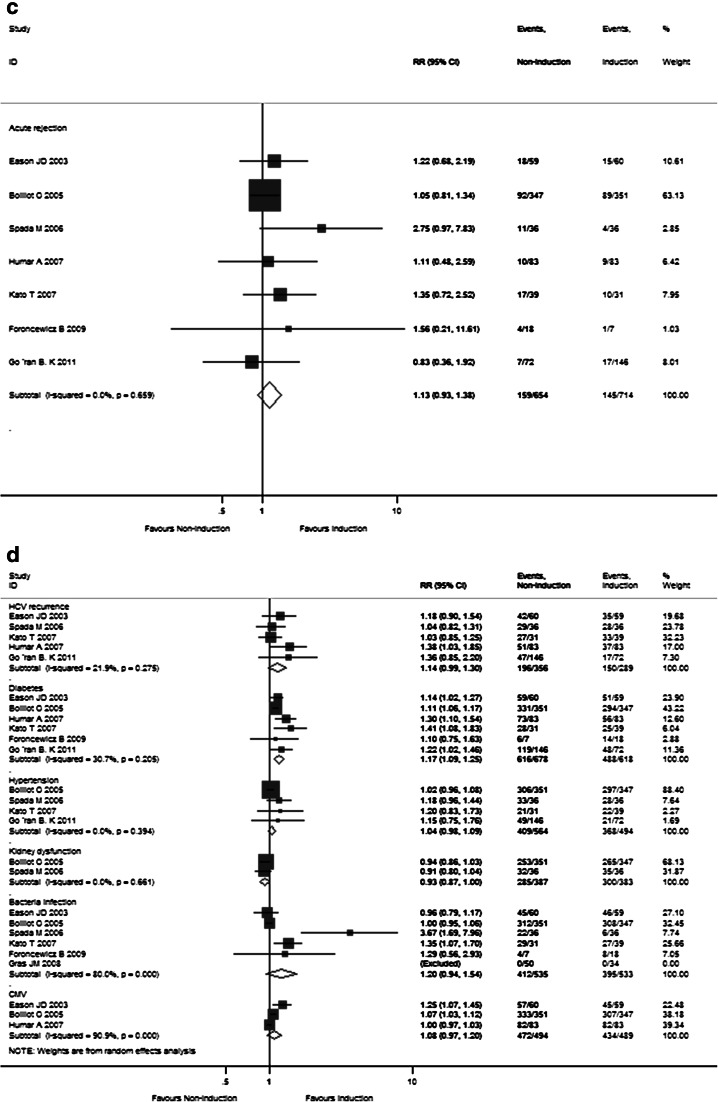
Forest plot of RR and 95 % CI for patient survival rates (**a**), graft survival rates (**b**), and rejection rates (**c**) and incidence of complications (**d**) for Sect. II in this study. The *horizontal lines* represent the 95 % CI of the RR for the non-induction group compared to the induction group in each study. The *black box* in the middle of the CI represents the single best estimate of RR in that study. The width of the CI is related to the power of the study and inversely associated with sample size. In addition, the pooled or combined RR results of the meta-analysis are represented by a *diamond*, the width of which is the CI for the pooled data. The *vertical line* is typically displayed to indicate no effect when RR = 1. When the CI crosses the *vertical line* of no effect, we must accept the null hypothesis of no difference between two groups. Only if the CI remains clear of the *vertical line* of no effect can we reject the null hypothesis and conclude that the prescription of induction agents during steroid minimization likely caused the outcome. We used a fixed effect model for meta-analysis, except that heterogeneity between studies was considered present if the *p* value was <0.1 or *I* ^*2*^ was more than 50 %, where we used a random effect model instead

The detailed pooled RR estimates of survival rates and rejection rates for Sects. I and II are listed in Supplementary Tables 3 and 4, respectively. In Sect. I, the overall 1-, 2-, 3-, and 5-year pooled RR estimates of patient survivals and graft survival rates were 0.988 (95 % CI 0.896–1.090), 1.032 (95 % CI 0.931–1.145), 1.021 (95 % CI 0.876–1.189), and 1.100 (95 % CI 0.968–1.250), as well as 0.991 (95 % CI 0.879–1.118), 1.013 (95 % CI 0.847–1.212), 0.905 (95 % CI 0.606–1.352), and 1.061 (95 % CI 0.855–1.316), respectively (Fig. 3a, b). The other pooled RR estimates of acute rejection and chronic rejection rates were 0.983 (95 % CI 0.774–1.247) and 0.126 (95 % CI 0.030–0.526) (Fig. 3c). In Sect. II, the overall 1- and 2-year pooled RR estimates of patient survival rates and graft survival rates were 0.982 (95 % CI 0.904–1.065) and 0.977 (95 % CI 0.895–1.067) as well as 1.005 (95 % CI 0.916–1.102) and 0.968 (95 % CI 0.863–1.085), respectively (Fig. 4a, b). The pooled RR estimate of acute rejection rates was 1.130 (95 % CI 0.927–1.377) (Fig. 4c). In general, steroid elimination and plus induction agent employment during OLT could achieve comparably favorable survival rates and rejection rates of no significance compared with traditional long-term steroid usage.

#### Secondary predictors

As shown in Supplementary Table 2 and Fig. 2d, the pooled RR estimates of secondary outcomes such as HCV recurrence (1.101; 95 % CI 0.964–1.257), HCC recurrence (1.403; 95 % CI 0.422–4.688), diabetes (1.836; 95 % CI 1.294–2.606), hypertension (1.607; 95 % CI 0.926–1.228), kidney dysfunction (0.842; 95 % CI 0.693–1.022), bacterial infection (1.096; 95 % CI 0.783–1.533), and CMV (2.280; 95 % CI 1.500–3.465) for all 17 RCTs were presented. Of note, the combined complication incidence estimates including diabetes (*p* = 0.001) and CMV (*p* < 0.001) were significantly reduced with early steroid withdrawal. In Sect. I, the pooled RR estimates of HCV recurrence (0.926; 95 % CI 0.586–1.463), HCC recurrence (2.437; 95 % CI 0.890–6.678), diabetes (1.223; 95 % CI 0.766–1.954), hypertension (0.975; 95 % CI 0.503–1.889), kidney dysfunction (0.807; 95 % CI 0.442–1.472), bacterial infection (0.529; 95 % CI 0.261–1.072), and CMV (2.137; 95 % CI 0.809–5.643) are displayed in Fig. 3d. No significance was observed between the steroid group and steroid-free group with respect to any complication incidence. In Sect. II, the pooled RR estimates of HCV recurrence (1.136; 95 % CI 0.993–1.300), diabetes (1.170; 95 % CI 1.093–1.252), hypertension (1.036; 95 % CI 0.980–1.095), kidney dysfunction (0.934; 95 % CI 0.869–1.004), bacterial infection (1.204; 95 % CI 0.941–1.541), and CMV (1.079; 95 % CI 0.968–1.203) are displayed in Fig. 4d. Compared with the non-induction group, the combined diabetes incidence estimates of the induction group were significantly decreased upon induction agent intervention (*p* < 0.001). The detailed pooled RR estimates of complication incidence for Sects. I and II are listed in Supplementary Tables 3 and 4, respectively.

### Publication bias

The funnel plot did not show any asymmetrical pattern, and the Begg’s test did not reveal any significant publication bias (data not shown).

## Discussion

To the best of our knowledge, our current study represents the first evidence-based medicine article concerning the efficacy and safety of steroid minimization in the Tac-based immunosuppressive regimen for liver transplant recipients. In this meta-analysis, altogether 17 clinical trials containing 1,980 transplanted patients published between 1995 and 2011 were finally enrolled in this study. To clarify whether induction agents could reduce potential adverse effects related to steroid avoidance by modulating the immunologic status, the enrolled studies were divided into two sections in terms of: (1) whether a steroid was employed upon OLT or not; (2) whether induction agents were employed during OLT or not. To our excitement, our results indicated that early steroid withdrawal or steroid avoidance in the Tac-based immunosuppressive regimen is safe and effective for the prevention of acute and chronic rejection after OLT with the benefit of a decrease in the incidence of diabetes and CMV infection. Although the underlying data remain to be systematically investigated, the present study revealed the introduction of some induction agents including RATG, basiliximab and daclizumab in triple and quadruple immunosuppressive protocols are likely more effective in lowering the high-dose usage of Tac in order to minimize the potential detrimental effect on renal functions. In the following paragraphs, some important issues pertaining to steroid elimination in the Tac-based immunosuppressive regimen for liver transplant recipients will be discussed.

Since the discovery of cortisol in 1937, steroids have paved the way for successful medical immunosuppression, especially for later organ transplantation, and proved the reversibility of rejection. However, in the modern era of an improved immunosuppressive protocol, it is necessary to critically assess the risk:benefit ratio of long-term steroid therapy during and after liver transplantation. To date, several studies have shown that weaning from steroids can be successfully carried out shortly after liver transplantation, thereby decreasing typical steroid-related side effects including new-onset diabetes mellitus, lipid metabolism abnormality, viral hepatitis recurrence and liver malignancy relapse [27, 29, 30, 35, 38]. In spite of these encouraging results, the theoretical advantages should be carefully balanced against the potential risks of increasing nonsteroidal immunosuppressive complications and a higher incidence of rejection. Jain et al. [39] reported 23.8 % of patients under the Tac-based immunosuppressive regimen required steroid reintroduction for late rejection, recurrence of the autoimmune process, renal impairment, or the concomitant presence of other medical conditions. Thus, the authors concluded that long-term sustained freedom from steroids may not be possible in all patients under Tac secondary to these conditions. Another multicenter, 1-year, comparative, double-blind, placebo-controlled study evaluating the efficacy and safety of an immunosuppressive regimen with steroid withdrawal at day 14 revealed a higher incidence of acute rejection, only balanced by a trend of a lower need for antidiabetic treatment [40]. The previous study suggested that steroid withdrawal or avoidance may not always be safe and needed. Steroid reintroduction may be necessary for late rejection episodes, recurrent autoimmune disease, or renal impairment due to Tac. As mentioned above, there are three categories of individuals in whom the long-term adverse effects of steroids after liver transplantation are particularly detrimental. First, most steroid-induced side effects occurred in children, like what was encountered in adults. Of note, growth retardation and Cushingoid features are of concern in the pediatric transplant recipients. Second, those patients with cardiovascular risk factors of hyperlipidemia, hypertension, or diabetes certainly have a contraindication to long-term steroid therapy. Last but not least, it could be supposed that rapid steroid tapering or being steroid free will serve as one of the most important determinants for slowing down the progression to tumor relapse.

For years corticosteroid induction has been the traditional standard immunosuppressive modality for OLT. Recently, induction therapy with antibodies has been increasingly used without widespread acceptance. The underlying mechanism of induction therapy is to inhibit thymus-derived lymphocyte activation through T cell pool depletion with either monoclonal antibodies or polyclonal antibodies (RATG), or to block specific IL-2 receptors (basiliximab or daclizumab), which may lead to reduction of the incidence of acute rejection and act as steroid-sparing minimization alternatives [19]. Mangus et al. [20] retrospectively analyzed data obtained from a single-center research database ranging from 2001 to 2008 comparing transplant outcomes and complications. The authors concluded that RATG-based induction immunosuppression could be safely used in adult OLT recipients with excellent survival, low rejection rates, and a comparably acceptable incidence of side effects [20]. Experts from the University of Tokyo in Japan conducted an observational study to evaluate the efficacy and safety of basiliximab as rescue therapy for the treatment of acute cellular rejection [41]. In contrast to 11 patients who received steroid therapy for acute cellular rejection, there were no significant immediate adverse effects in the basiliximab group which underwent liver transplantation for HCV cirrhosis [41]. In addition, recent studies have shown that immunosuppression with low-dose daclizumab and delayed initiation of Tac had significant benefits in preserving renal function after OLT [42]. However, the application of the induction therapy with biologic agents carrying elevated risks of over-immunosuppression, CMV viremia, posttransplant lymphoproliferative disease, as well as HCV recurrence is still controversial [43]. In our present study, we have demonstrated comparable patient and graft survival with significantly lower rates of HCV recurrence, diabetes, bacterial infection, and CMV infection as compared to no induction intervention.

## Conclusions

In conclusion, the present investigation systematically reviewed the recent 17 prospective randomized controlled clinical trials concerning the application of steroid minimization in the Tac-based immunosuppressive regimen for liver transplant recipients, and a meta-analysis was performed to reveal the efficacy and safety of early steroid withdrawal or complete avoidance. Furthermore, adverse events potentially related to steroids were less frequently observed with the use of antibody agents for induction therapy while low-dose Tac could be maintained.

## Electronic supplementary material

Below is the link to the electronic supplementary material.

Supplementary material 1 (DOC 45 kb)

Supplementary material 2 (DOC 44 kb)

Supplementary material 3 (DOC 45 kb)

Supplementary material 4 (DOC 39 kb)

Supplementary Fig. 5 Representative funnel plot of 1-year patient survival rate in Sect. I (TIFF 3051 kb)

## Abbreviations

AIH: Autoimmune hepatitis
CI: Confidence intervals
CMV: Cytomegalovirus
EBV: Epstein–Barr virus
HBV: Hepatitis B virus
HCC: Hepatocellular carcinoma
HCV: Hepatitis C virus
MELD: Model for end-stage liver disease
MMF: Mycophenolate mofetil
OLT: Orthotopic liver transplantation
PBC: Primary biliary cirrhosis
PSC: Primary sclerosing cholangitis
RATG: Rabbit antithymocyte globulin
RCT: Randomized controlled trials
RR: Risk ratios
Tac: Tacrolimus

## References

[1] Merion RM (2010). Current status and future of liver transplantation. Semin Liver Dis.

[2] Sánchez-Fueyo A, Strom TB (2011). Immunologic basis of graft rejection and tolerance following transplantation of liver or other solid organs. Gastroenterology.

[3] Levitsky J (2011). Operational tolerance: past lessons and future prospects. Liver Transpl.

[4] Fändrich F (2011). Tolerance in clinical transplantation: progress, challenge or just a dream?. Langenbecks Arch Surg.

[5] Salcedo M, Vaquero J, Bañares R, Rodríguez-Mahou M, Alvarez E, Vicario JL (2002). Response to steroids in de novo autoimmune hepatitis after liver transplantation. Hepatology.

[6] Gruttadauria S, di Francesco F, Pagano D, Vizzini G, Cintorino D, Spada M (2011). Complications in immunosuppressive therapy of liver transplant recipients. J Surg Res.

[7] Bhat I, Mukherjee S (2009). Hepatitis C recurrence after liver transplantation. Panminerva Med.

[8] Ciesek S, Steinmann E, Iken M, Ott M, Helfritz FA, Wappler I (2010). Glucocorticosteroids increase cell entry by hepatitis C virus. Gastroenterology.

[9] Chen ZS, He F, Zeng FJ, Jiang JP, Du DF, Liu B (2007). Early steroid withdrawal after liver transplantation for hepatocellular carcinoma. World J Gastroenterol.

[10] Park JW, Lee KW, Kim SJ, Choi SH, Heo JS, Kwon CH (2006). Outcome of patients with recurrent hepatocellular carcinoma in liver transplantation. Transplant Proc.

[11] Pirenne J, Aerts R, Koshiba T, Van Gelder F, Roskams T, Schetz M (2003). Steroid-free immunosuppression during and after liver transplantation—a 3-yr follow-up report. Clin Transplant.

[12] Ringe B, Braun F, Schütz E, Füzesi L, Lorf T, Canelo R (2001). A novel management strategy of steroid-free immunosuppression after liver transplantation: efficacy and safety of tacrolimus and mycophenolate mofetil. Transplantation.

[13] Diem HV, Sokal EM, Janssen M, Otte JB, Reding R (2003). Steroid withdrawal after pediatric liver transplantation: a long-term follow-up study in 109 recipients. Transplantation.

[14] Reding R, Gras J, Sokal E, Otte JB, Davies HF (2003). Steroid-free liver transplantation in children. Lancet.

[15] Samonakis DN, Mela M, Quaglia A, Triantos CK, Thalheimer U, Leandro G (2006). Rejection rates in a randomised trial of tacrolimus monotherapy versus triple therapy in liver transplant recipients with hepatitis C virus cirrhosis. Transpl Infect Dis.

[16] Moench C, Barreiros AP, Schuchmann M, Bittinger F, Thiesen J, Hommel G (2007). Tacrolimus monotherapy without steroids after liver transplantation—a prospective randomized double—blinded placebo-controlled trial. Am J Transplant.

[17] Reggiani P, Arru M, Regazzi M, Gatti S, Molinaro MD, Caccamo L (2005). A steroid-free tacrolimus and low-dose mycophenolate mofetil primary immunosuppression does not prevent early acute rejection after liver transplantation. Transplant Proc.

[18] Foroncewicz B, Mucha K, Ryszkowska E, Ciszek M, Ziółkowski J, Porowski D (2009). Safety and Efficacy of steroid-free immunosuppression with tacrolimus and daclizumab in liver transplant recipients: 6-year follow-up in a single center. Transplant Proc.

[19] Figueras J, Prieto M, Bernardos A, Rimola A, Suárez F, de Urbina JO (2006). Daclizumab induction and maintenance steroid-free immunosuppression with mycophenolate mofetil and tacrolimus to prevent acute rejection of hepatic allografts. Transpl Int.

[20] Mangus RS, Fridell JA, Vianna RM, Kwo PY, Chen J, Tector AJ (2012). Immunosuppression induction with rabbit antithymocyte globulin (rATG) ± rituximab in 1000 liver transplants with long-term follow-up. Liver Transpl.

[21] Sato K, Sekiguchi S, Kawagishi N, Akamatsu Y, Ishida K, Fukushima D (2011). Unique histopathological features of graft biopsies with liver function abnormalities in living donor liver transplant patients receiving basiliximab induction therapy. Clin Transplant.

[22] Moher D, Liberati A, Tetzlaff J, Altman DG, Group PRISMA (2009). Preferred reporting items for systematic reviews and meta-analyses: the PRISMA statement. PLoS Med.

[23] Higgins JPT, Green S. Cochrane Handbook for Systematic Reviews of Interventions. Version 5.0.1. The Cochrane Collaboration; 2008. www.cochrane-handbook.org. Accessed 2 July 2009

[24] Higgins JPT, Altman DG, Sterne JAC. Chapter 8: Assessing risk of bias in included studies. In: Higgins JPT, Green S, editors. Cochrane Handbook for Systematic Reviews of Interventions Version 5.1.0 (updated March 2011). The Cochrane Collaboration 2011. http://www.cochrane-handbook.org. Accessed 1 July 2012

[25] Langrehr JM, Neumann UP, Lang M, Müller AR, Jonas S, Settmacher U (2002). First results from a prospective randomized trial comparing steroid-free induction therapy with tacrolimus and MMF versus tacrolimus and steroids in patients after liver transplantation for HCV. Transplant Proc.

[26] Pelletier SJ, Vanderwall K, Debroy MA, Englesbe MJ, Sung RS, Magee JC (2005). Preliminary analysis of early outcomes of a prospective, randomized trial of complete steroid avoidance in liver transplantation. Transplant Proc.

[27] Margarit C, Bilbao I, Castells L, Lopez I, Pou L, Allende E (2005). A prospective randomized trial comparing tacrolimus and steroids with tacrolimus monotherapy in liver transplantation: the impact on recurrence of hepatitis C. Transpl Int.

[28] Junge G, Neuhaus R, Schewior L, Klupp J, Guckelberger O, Langrehr JM (2005). Withdrawal of steroids: a randomized prospective study of prednisone and tacrolimus versus mycophenolate mofetil and tacrolimus in liver transplant recipients with autoimmune hepatitis. Transplant Proc.

[29] Vivarelli M, Burra P, La Barba G, Canova D, Senzolo M, Cucchetti A (2007). Influence of steroids on HCV recurrence after liver transplantation: a prospective study. J Hepatol.

[30] Manousou P, Samonakis D, Cholongitas E, Patch D, O’Beirne J, Dhillon AP (2009). Outcome of recurrent hepatitis C virus after liver transplantation in a randomized trial of tacrolimus monotherapy versus triple therapy. Liver Transpl.

[31] Weiler N, Thrun I, Hoppe-Lotichius M, Zimmermann T, Kraemer I, Otto G (2010). Early steroid-free immunosuppression with FK506 after liver transplantation: long-term results of a prospectively randomized double-blinded trial. Transplantation.

[32] Eason JD, Nair S, Cohen AJ, Blazek JL, Loss GE (2003). Steroid-free liver transplantation using rabbit antithymocyte globulin and early tacrolimus monotherapy. Transplantation.

[33] Boillot O, Mayer DA, Boudjema K, Salizzoni M, Gridelli B, Filipponi F (2005). Corticosteroid-free immunosuppression with tacrolimus following induction with daclizumab: a large randomized clinical study. Liver Transpl.

[34] Spada M, Petz W, Bertani A, Riva S, Sonzogni A, Giovannelli M (2006). Randomized trial of basiliximab induction versus steroid therapy in pediatric liver allograft recipients under tacrolimus immunosuppression. Am J Transplant.

[35] Humar A, Crotteau S, Gruessner A, Kandaswamy R, Gruessner R, Payne W (2007). Steroid minimization in liver transplant recipients: impact on hepatitis C recurrence and post-transplant diabetes. Clin Transplant.

[36] Kato T, Gaynor JJ, Yoshida H, Montalvano M, Takahashi H, Pyrsopoulos N (2007). Randomized trial of steroid-free induction versus corticosteroid maintenance among orthotopic liver transplant recipients with hepatitis C virus: impact on hepatic fibrosis progression at one year. Transplantation.

[37] Gras JM, Gerkens S, Beguin C, Janssen M, Smets F, Otte JB (2008). Steroid-free, tacrolimus-basiliximab immunosuppression in pediatric liver transplantation: clinical and pharmacoeconomic study in 50 children. Liver Transpl.

[38] Klintmalm GB, Davis GL, Teperman L, Netto GJ, Washburn K, Rudich SM (2011). A randomized, multicenter study comparing steroid-free immunosuppression and standard immunosuppression for liver transplant recipients with chronic hepatitis C. Liver Transpl.

[39] Jain A, Kashyap R, Marsh W, Rohal S, Khanna A, Fung JJ (2001). Reasons for long-term use of steroid in primary adult liver transplantation under tacrolimus. Transplantation.

[40] Pageaux GP, Calmus Y, Boillot O, Ducerf C, Vanlemmens C, Boudjema K (2004). Steroid withdrawal at day 14 after liver transplantation: a double-blind, placebo-controlled study. Liver Transpl.

[41] Togashi J, Sugawara Y, Tamura S, Kaneko J, Yamashiki N, Aoki T (2011). Basiliximab as therapy for acute rejection after liver transplantation for hepatitis C virus cirrhosis. Biosci Trends.

[42] Post M, Raszeja-Wyszomirska J, Jarosz K, Lubikowski J, Wasilewicz M, Mydłowska M (2009). Immunosuppression with low-dose daclizumab in liver transplant recipients with impaired kidney function: a single-center experience. Transplant Proc.

[43] Ramirez CB, Marino IR (2007). The role of basiliximab induction therapy in organ transplantation. Expert Opin Biol Ther.

