# Supplementary material for: Role of steroid minimization in the tacrolimus-based immunosuppressive regimen for liver transplant recipients: a systematic review and meta-analysis of prospective randomized controlled trials
Source: Hepatol Int. 2014 Mar 20;8(2):198–215. doi: 10.1007/s12072-014-9523-y (PMC3990862; doi:10.1007/s12072-014-9523-y)
Supplement: Supplementary file 1 — Supplementary material 1 (DOC 45 kb) [file 12072_2014_9523_MOESM1_ESM.doc]

**Supplementary Table 1 Quality of each RCT included in the meta-analysis**

| **First author** | **Randomization** | **Patients blinding** | **Concealed allocation** | **Intention-to-treat analysis** | **Incomplete outcome** |
| --- | --- | --- | --- | --- | --- |
| [Langrehr JM](http://www.ncbi.nlm.nih.gov/pubmed?term="Langrehr JM"[Author]) [25] | Yes | Unclear | Unclear | Unclear | Yes |
| [Eason JD](http://www.ncbi.nlm.nih.gov/pubmed?term="Eason JD"[Author]) [32] | Yes | Unclear | Unclear | Unclear | Yes |
| [Pelletier SJ](http://www.ncbi.nlm.nih.gov/pubmed?term="Pelletier SJ"[Author]) [26] | Yes | Unclear | Unclear | Yes | Yes |
| [Margarit C](http://www.ncbi.nlm.nih.gov/pubmed?term="Margarit C"[Author]) [27] | Yes | Unclear | Unclear | Unclear | No |
| [Reggiani P](http://www.ncbi.nlm.nih.gov/pubmed?term="Reggiani P"[Author]) [17] | Yes | Unclear | Unclear | Unclear | Yes |
| [Junge G](http://www.ncbi.nlm.nih.gov/pubmed?term="Junge G"[Author]) [28] | Yes | Unclear | Unclear | Unclear | Yes |
| [Boillot O](http://www.ncbi.nlm.nih.gov/pubmed?term="Boillot O"[Author]) [33] | Yes | Unclear | Unclear | Yes | Yes |
| [Spada M](http://www.ncbi.nlm.nih.gov/pubmed?term="Spada M"[Author]) [34] | Yes | Unclear | Unclear | Unclear | Yes |
| Chen ZS [9] | Yes | Unclear | Unclear | Unclear | Yes |
| [Vivarelli M](http://www.ncbi.nlm.nih.gov/pubmed?term="Vivarelli M"[Author]) [29] | Yes | Unclear | Unclear | Unclear | Yes |
| [Humar A](http://www.ncbi.nlm.nih.gov/pubmed?term="Humar A"[Author]) [35] | Yes | Unclear | Unclear | Unclear | Yes |
| [Kato T](http://www.ncbi.nlm.nih.gov/pubmed?term="Kato T"[Author]) [36] | Yes | Unclear | Unclear | Unclear | Yes |
| [Gras JM](http://www.ncbi.nlm.nih.gov/pubmed?term="Gras JM"[Author]) [37] | Yes | Unclear | Unclear | Unclear | Yes |
| [Manousou P](http://www.ncbi.nlm.nih.gov/pubmed?term="Manousou P"[Author]) [30] | Yes | Yes | Yes | Yes | Yes |
| [Foroncewicz B](http://www.ncbi.nlm.nih.gov/pubmed?term="Foroncewicz B"[Author]) [18] | Yes | Unclear | Unclear | Unclear | Yes |
| [Weiler N](http://www.ncbi.nlm.nih.gov/pubmed?term="Weiler N"[Author]) [31] | Yes | Yes | Yes | Unclear | No |
| [Klintmalm GB](http://www.ncbi.nlm.nih.gov/pubmed?term=Klintmalm GB%5BAuthor%5D&cauthor=true&cauthor_uid=21850690) [38] | Yes | Unclear | Unclear | Yes | Yes |
