# Supplementary material for: Role of steroid minimization in the tacrolimus-based immunosuppressive regimen for liver transplant recipients: a systematic review and meta-analysis of prospective randomized controlled trials
Source: Hepatol Int. 2014 Mar 20;8(2):198–215. doi: 10.1007/s12072-014-9523-y (PMC3990862; doi:10.1007/s12072-014-9523-y)
Supplement: Supplementary file 2 — Supplementary material 2 (DOC 44 kb) [file 12072_2014_9523_MOESM2_ESM.doc]

**Supplementary Table 2 Meta-analysis results of pooled outcomes including primary and secondary endpoints for all 17 enrolled RCT in this study**

| **Observational outcomes** | **Pooled RR** | **95%*CI*** | ***p* value** | **P value** | **I^2^ (%)** |
| --- | --- | --- | --- | --- | --- |
| ***Primary endpoints*** | | | | | |
| 1-year patient survival | 0.985 | 0.925, 1.048 | 0.627 | 0.793 | 0.0 |
| 2-year patient survival | 0.998 | 0.934, 1.067 | 0.961 | 0.829 | 0.0 |
| 3-year patient survival | 0.995 | 0.894, 1.107 | 0.921 | 0.567 | 0.0 |
| 5-year patient survival | 1.100 | 0.968, 1.250 | 0.143 | 0.811 | 0.0 |
| 1-year graft survival | 0.998 | 0.928, 1.072 | 0.946 | 0.614 | 0.0 |
| 2-year graft survival | 0.993 | 0.902, 1.092 | 0.877 | 0.483 | 0.0 |
| 3-year graft survival | 0.945 | 0.833, 1.072 | 0.376 | 0.129 | 51.1 |
| 5-year graft survival | 1.053 | 0.849, 1.307 | 0.638 | 0.416 | 0.0 |
| Acute rejection | 1.077 | 0.864, 1.343 | 0.510 | 0.100 | 32.8 |
| Chronic rejection | 0.311 | 0.003, 37.207 | 0.632 | 0.022 | 80.9 |
| ***Secondary endpoints*** | | | | | |
| HCV recurrence | 1.101 | 0.964, 1.257 | 0.155 | 0.258 | 19.5 |
| HCC recurrence | 1.403 | 0.422, 4.688 | 0.581 | 0.260 | 21.1 |
| Diabetes | 1.836 | 1.294, 2.606 | 0.001 | 0.029 | 48.6 |
| Hypertension | 1.607 | 0.926, 1.228 | 0.369 | 0.495 | 0.0 |
| Kidney dysfunction | 0.842 | 0.693, 1.022 | 0.083 | 0.664 | 0.0 |
| Bacteria infection | 1.096 | 0.783, 1.533 | 0.594 | 0.002 | 67.2 |
| CMV | 2.280 | 1.500, 3.465 | <0.001 | 0.566 | 0.0 |

*CI*: confidence interval; RR: risk ratio.
