# Supplementary material for: Role of steroid minimization in the tacrolimus-based immunosuppressive regimen for liver transplant recipients: a systematic review and meta-analysis of prospective randomized controlled trials
Source: Hepatol Int. 2014 Mar 20;8(2):198–215. doi: 10.1007/s12072-014-9523-y (PMC3990862; doi:10.1007/s12072-014-9523-y)
Supplement: Supplementary file 3 — Supplementary material 3 (DOC 45 kb) [file 12072_2014_9523_MOESM3_ESM.doc]

**Supplementary Table 3 Meta-analysis results of pooled outcomes including primary and secondary endpoints for Section I in this study**

| **Observational outcomes** | **Pooled RR** | **95%*CI*** | ***p* value** | **P value** | **I^2^ (%)** |
| --- | --- | --- | --- | --- | --- |
| ***Primary endpoints*** | | | | | |
| 1-year patient survival | 0.988 | 0.896, 1.090 | 0.817 | 0.614 | 0.0 |
| 2-year patient survival | 1.032 | 0.931, 1.145 | 0.547 | 0.702 | 0.0 |
| 3-year patient survival | 1.021 | 0.876, 1.189 | 0.793 | 0.498 | 0.0 |
| 5-year patient survival | 1.100 | 0.968, 1.250 | 0.143 | 0.811 | 0.0 |
| 1-year graft survival | 0.991 | 0.879, 1.118 | 0.887 | 0.341 | 7.1 |
| 2-year graft survival | 1.013 | 0.847, 1.212 | 0.884 | 0.279 | 14.5 |
| 3-year graft survival | 0.905 | 0.606, 1.352 | 0.627 | 0.044 | 75.4 |
| 5-year graft survival | 1.061 | 0.855, 1.316 | 0.590 | 0.416 | 0.0 |
| Acute rejection | 0.983 | 0.774, 1.247 | 0.886 | 0.019 | 56.2 |
| Chronic rejection | 0.126 | 0.030, 0.526 | 0.004 | 0.022 | 80.9 |
| ***Secondary endpoints*** | | | | | |
| HCV recurrence | 0.926 | 0.586, 1.463 | 0.742 | 0.305 | 16.8 |
| HCC recurrence | 2.437 | 0.890, 6.678 | 0.083 | 0.149 | 52.0 |
| Diabetes | 1.223 | 0.766, 1.954 | 0.400 | 0.231 | 27.1 |
| Hypertension | 0.975 | 0.503, 1.889 | 0.940 | 0.413 | 0.0 |
| Kidney dysfunction | 0.807 | 0.442, 1.472 | 0.484 | 0.437 | 0.0 |
| Bacteria infection | 0.529 | 0.261, 1.072 | 0.077 | 0.294 | 19.2 |
| CMV | 2.137 | 0.809, 5.643 | 0.125 | 0.395 | 0.0 |

*CI*: confidence interval; OR: risk ratio.
