# Supplementary material for: Role of steroid minimization in the tacrolimus-based immunosuppressive regimen for liver transplant recipients: a systematic review and meta-analysis of prospective randomized controlled trials
Source: Hepatol Int. 2014 Mar 20;8(2):198–215. doi: 10.1007/s12072-014-9523-y (PMC3990862; doi:10.1007/s12072-014-9523-y)
Supplement: Supplementary file 4 — Supplementary material 4 (DOC 39 kb) [file 12072_2014_9523_MOESM4_ESM.doc]

**Supplementary Table 4 Meta-analysis results of pooled outcomes including primary and secondary endpoints for Section II in this study**

| **Observational outcomes** | **Pooled RR** | **95%*CI*** | ***p* value** | **P value** | **I^2^ (%)** |
| --- | --- | --- | --- | --- | --- |
| ***Primary endpoints*** | | | | | |
| 1-year patient survival | 0.982 | 0.904, 1.065 | 0.658 | 0.599 | 0.0 |
| 2-year patient survival | 0.977 | 0.895, 1.067 | 0.607 | 0.555 | 0.0 |
| 1-year graft survival | 1.005 | 0.916, 1.102 | 0.915 | 0.503 | 0.0 |
| 2-year graft survival | 0.968 | 0.863, 1.085 | 0.576 | 0.279 | 14.8 |
| Acute rejection | 1.130 | 0.927, 1.377 | 0.228 | 0.659 | 0.0 |
| ***Secondary endpoints*** | | | | | |
| HCV recurrence | 1.136 | 0.993, 1.300 | 0.063 | 0.275 | 21.9 |
| Diabetes | 1.170 | 1.093, 1.252 | <0.001 | 0.205 | 30.7 |
| Hypertension | 1.036 | 0.980, 1.095 | 0.211 | 0.394 | 0.0 |
| Kidney dysfunction | 0.934 | 0.869, 1.004 | 0.065 | 0.661 | 0.0 |
| Bacteria infection | 1.204 | 0.941, 1.541 | 0.141 | <0.001 | 80.0 |
| CMV | 1.079 | 0.968, 1.203 | 0.170 | <0.001 | 90.9 |

*CI*: confidence interval; OR: risk ratio.
