# Supplementary figures and images for: Role of steroid minimization in the tacrolimus-based immunosuppressive regimen for liver transplant recipients: a systematic review and meta-analysis of prospective randomized controlled trials
Source: Hepatol Int. 2014 Mar 20;8(2):198–215. doi: 10.1007/s12072-014-9523-y (PMC3990862; doi:10.1007/s12072-014-9523-y)

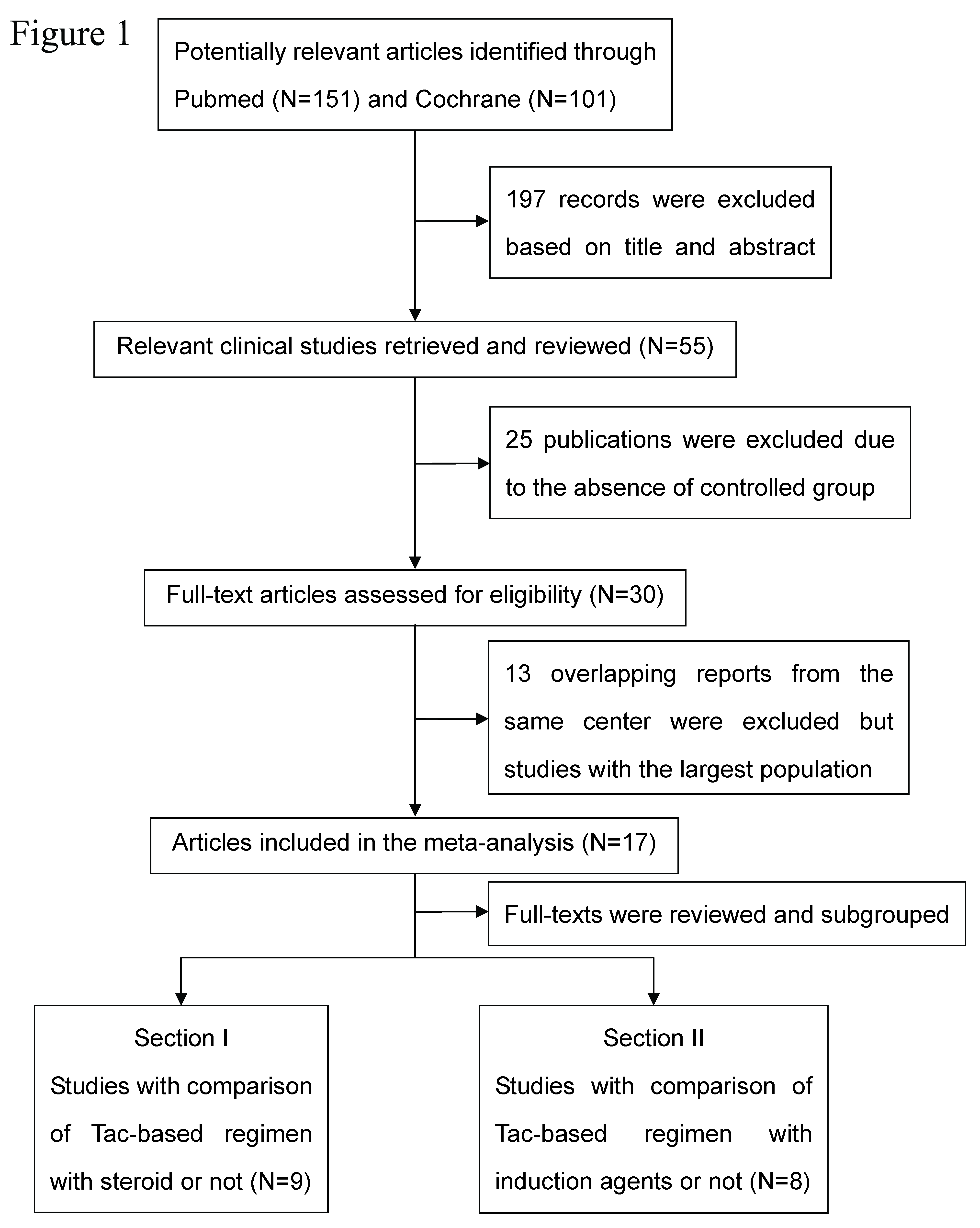

Supplement: Supplementary file 5 — Representative funnel plot of 1-year patient survival rate in Sect. I (TIFF 3051 kb) [file 12072_2014_9523_MOESM5_ESM.tif]
